# Closest Farthest Widest

**DOI:** 10.3390/a17030095

**Published:** 2024-02-22

**Authors:** Kenneth Lange

**Affiliations:** Departments of Computational Medicine, Human Genetics, and Statistics, University of California, Los Angeles, CA 90095, USA

**Keywords:** convex set, diameter, farthest point, Frank-Wolfe, homotopy, projected gradient ascent

## Abstract

The current paper proposes and tests algorithms for finding the diameter of a compact convex set and the farthest point in the set to another point. For these two nonconvex problems, I construct Frank–Wolfe and projected gradient ascent algorithms. Although these algorithms are guaranteed to go uphill, they can become trapped by local maxima. To avoid this defect, I investigate a homotopy method that gradually deforms a ball into the target set. Motivated by the Frank–Wolfe algorithm, I also find the support function of the intersection of a convex cone and a ball centered at the origin and elaborate a known bisection algorithm for calculating the support function of a convex sublevel set. The Frank–Wolfe and projected gradient algorithms are tested on five compact convex sets: (a) the box whose coordinates range between −1 and 1, (b) the intersection of the unit ball and the non-negative orthant, (c) the probability simplex, (d) the Manhattan-norm unit ball, and (e) a sublevel set of the elastic net penalty. Frank–Wolfe and projected gradient ascent are about equally fast on these test problems. Ignoring homotopy, the Frank–Wolfe algorithm is more reliable. However, homotopy allows projected gradient ascent to recover from its failures.

## 1. Introduction

Let S be a compact convex set and p any external or internal point. This paper investigates algorithms for computing the two functions ${far}_{S}(p)={max}_{x\in S}\|x-p\|$ and $diam(S)={max}_{(x,y)\in S\times S}\|x-y\|$. The first is the farthest distance from p to x∈S, and the second is the diameter of S. Both objective functions are convex and Lipschitz under the Euclidean norm, the first with constant 1 and the second with constant 2. Furthermore, if S is merely bounded, and conv(S) denotes its closed convex hull, and ext(S) denotes the extreme points of conv(S), then

$$\begin{array}{l} \;{far}_{S}(p)={far}_{conv(S)}(p)={far}_{ext(S)}(p)\\ diam(S)=diam\left[conv\left(S\right)\right]=diam\left[ext\left(S\right)\times ext\left(S\right)\right]. \end{array}$$

The fact that conv(S) is compact is crucial in the reaching these conclusions. Thus, without loss of generality, we can assume that S is a compact convex set. All boundary points S are extreme points when S is strictly convex [1,2].

If S is the convex hull of a finite cloud of points $\left\{x_{1},\ldots ,x_{m}\right\}$, then ${far}_{S}(p)$ can be found by identifying the point $x_{i}$ that maximizes $\left\|x_{i}-p\right\|$. For a ball S and a point y∉S, ${far}_{S}(p)$ is found by projecting y onto S and then extending the line through y and its projection z to the antipodal point of z. If the set S is a Cartesian product, then calculation of ${far}_{S}(p)$ and diam(S) reduce to easy lower-dimensional problems. Many diameters are known. For a ball, ellipsoid $\left\{x:\frac{1}{2}x^{⊤}Ax\leq r\right\}$, rectangle [a,b], probability simplex, and $\ell_{1}$ unit ball, the diameter is, respectively, twice its radius, $\sqrt{8r\lambda_{min}^{-1}},\;\|a-b\|$, $\sqrt{2}$, and 2 , where $\lambda_{min}$ is the smallest eigenvalue of the positive definite matrix A [3].

The distance function $dist(p,S)={min}_{x\in S}\|x-p\|$ is extremely well studied [4–7]. The unique point where the minimum is attained is the projection $P_{S}(p)$ of p onto S. Many projection operators are known [4,6–9]. The web sites [10,11] provide Julia, Python, and Matlab implementations of the most commonly encountered projection operators. Projection onto a convex sublevel set S={x:g(x)≤0} is often more computationally demanding than other projection problems. For the special case when the proximal operator ${prox}_{\lambda g}(y)={argmin}_{x}\left[\lambda g(x)+\frac{1}{2}\|y-x{\|}^{2}\right]$ is known for all λ>0, one can solve the projection problem by bisection [12]. Bisection also turns out to be an attractive strategy for computing ${far}_{S}(p)$ and diam(S) for sublevel sets.

The corresponding operators for ${far}_{S}(p)$ and diam(S) can be multi-valued. Finding them is more challenging because the underlying optimization problem is no longer convex. The current paper proposes two algorithms for each problem. These algorithms are not infallible. They are ascent algorithms, so they tend to find local maxima. Convex hull algorithms can be harnessed to solve the farthest and diameter problems for finite point clouds in dimensions greater than 3 [13,14]. To our knowledge, these are the only competitive algorithms in common use in higher dimensions. Hence, any progress in solving these two fundamental geometric problems should be welcome.

Unfortunately, convex hull algorithms scale poorly in high dimensions. For n points in $R^{p}$, an n-vertex polytope can have as many as $O(n^{\left\lfloor \frac{p}{2}\right\rfloor })$ facets [15]. This translates into a worst-case computational computational complexity of $O(n^{\left\lfloor \frac{p}{2}\right\rfloor })$ for traditional convex hull algorithms. It is true that once the m extreme points are extracted from the convex hull, the farthest and diameter problems require computing just m and $m^{2}$ distances, respectively. In contrast, we circumvent the convex hull problem entirely and attack the farthest and diameter problems directly. Thus, we easily handle problems in dimension p=1000.

Given the naturalness of the farthest and diameter problems, a few comments should suffice to motivate each. The minimum enclosing ball problem reduces to find

$$\underset{c\in S}{argmin}\;\underset{x\in S}{max}\|x-c\|=\underset{c\in S}{argmin}\;\underset{S}{far}(c).$$

The solution point c is called the Chebyshev center. Article [16] explains the pertinence to statistics. As for the diameter problem, convergence estimates for the Frank–Wolfe method of optimization depend on the squared diameter of the underlying set [17,18]. In general for an L-Lipschitz function f(x), the inequality $\left|f\left(x\right)\right|\leq \left|f\left(y\right)\right|+L\;diam(S)$ bounds objective values. Bounding objective values is crucial in deriving complexity estimates for optimization algorithms such as projected gradient descent. If instead ∇f(x) possesses an L-Lipschitz gradient, then the bound

$$\left\|\nabla f\left(x\right)-\nabla f\left(y\right)\right\|\leq L\;diam(S)$$

is available for proving convergence to a stationary point. The diameter of a set also plays a crucial role in estimating the concentration of probability measures [19]. Finally, let us draw attention to a theorem of Jung to the effect that the radius r of the minimum enclosing ball problem satisfies $r\leq diam(S)\sqrt{\frac{p}{2(p+1)}}$ for $S\subset R^{p}$ [20].

Our algorithms are minorization–maximization (MM) algorithms [7,21]. Such algorithms depend on a surrogate function that minorizes the original objective f(x) around the current iterate $x_{n}$ in the sense of satisfying the tangency condition $g\left(x_{n}|x_{n}\right)=f\left(x_{n}\right)$ and the domination condition $g\left(x|x_{n}\right)\leq f(x)$ for all x. The surrogate balances two goals, hugging the objective tightly and simplifying maximization. Maximizing the surrogate produces the next iterate $x_{n+1}$ and drives the objective uphill because

$$f\left(x_{n+1}\right)\geq g\left(x_{n+1}|x_{n}\right)\geq g\left(x_{n}|x_{n}\right)=f\left(x_{n}\right).$$

In minimization, the surrogate majorizes the objective and is instead minimized. The tangency condition remains the same, but now the domination condition $g\left(x|x_{n}\right)\geq f(x)$ is reversed. The acronym MM also applies to majorization-minimization.

The celebrated EM (expectation–maximization) principle for maximum likelihood estimation with missing data [22] is a special case of minorization–maximization. In the EM setting, Jensen’s inequality supplies the surrogate as the expectation of the complete data log-likelihood conditional on the observed data. Projected gradient descent, proximal gradient descent, and the convex–concave procedure [23] can also be viewed as MM algorithms.

The convexity of $\frac{1}{2}\|x-p{\|}^{2}$ yields the supporting hyperplane minorization

$$\frac{1}{2}\|x-p{\|}^{2}\geq \frac{1}{2}{\left\|x_{n}-p\right\|}^{2}+{\left(x_{n}-p\right)}^{⊤}\left(x-x_{n}\right).$$

To improve $\|x-p{\|}^{2}$, we take

$$x_{n+1}\in \underset{x\in S}{argmax}\;v^{⊤}x$$

for $v=x_{n}-p$. This is the (naive) Frank-Wolfe algorithm [17,24] with full steps operating on the support function $\sigma_{S}(v)={sup}_{x\in S}v^{⊤}x$ defined by S. The collection of points ${supp}_{S}(v)={argmin}_{x\in S}v^{⊤}x$ at which the maximum is attained constitute the support map. Let us emphasize that the Frank–Wolfe algorithm is pertinent to the maximization of any differentiable convex function f(x) over a compact convex set S because the supporting hyperplane minorization

$$f(x)\geq f\left(x_{n}\right)+df\left(x_{n}\right)\left(x-x_{n}\right)$$

generalizes to this context. The ascent property follows immediately from this minorization and the MM principle.

The Frank–Wolfe method applies to a host of problems. For example, suppose g(x) maps $R^{p}$ into itself. Consider the problem of minimizing the loss $f(x)=\frac{1}{2}{\left\|g(x)\right\|}^{2}$ over a compact convex set S. The function f(x) has differential $df(x)=g{\left(x\right)}^{⊤}dg(x)$ and gradient $\nabla f(x)=dg{\left(x\right)}^{⊤}g(x)$. At iteration n, Frank–Wolfe decreases the linear approximation

$$f(y)\approx f\left(x_{n}\right)+g{\left(x_{n}\right)}^{⊤}dg\left(x_{n}\right)\left(y-x_{n}\right)$$

to f(y). If the support function of S is simple to compute, then Frank–Wolfe additionally needs to compute just a single matrix times vector product $dg{\left(x\right)}^{⊤}g(x)$ per iteration. The projection operator $P_{S}(x)$ is never invoked in updating x. In this minimization example, the MM principle is no longer operative, so a more sophisticated choice of step length is advisable. The problem of finding a root of g(x)=0 on S also succumbs to a conjugate gradient method [25].

There are many known examples of support functions. All are closed, convex, positively homogeneous, and satisfy $\sigma_{S}(0)=0$. The support function $\sigma_{S}(y)$ is by definition the Fenchel conjugate ${sup}_{x}\left[y^{⊤}x-i_{S}(x)\right]$ of the 0/∞ indicator $i_{S}(y)$ of S. It is worth mentioning a few known support functions. The support function of a closed convex cone is the convex indicator of its polar cone. The support function of a polyhedral set can be found by linear programming. The support function of a union of two sets is the maximum of the two support functions. The support function of a Minkowski sum is the sum of the two support functions. The support function of the unit ball of a norm is the dual norm. The support function of a Cartesian product is the sum of the support functions of the two product sets.

The Frank–Wolfe version of the diameter problem exploits the two supporting hyperplane minorizations

$$\begin{array}{ll} {\left\|x-y\right\|}^{2} & \geq {\left\|x_{n}-y_{n}\right\|}^{2}+2{\left(x_{n}-y_{n}\right)}^{⊤}\left(x-x_{n}\right)\\ {\left\|x-y\right\|}^{2} & \geq {\left\|x_{n}-y_{n}\right\|}^{2}+2{\left(y_{n}-x_{n}\right)}^{⊤}\left(y-y_{n}\right). \end{array}$$

Adding these and dividing by 2 yields the minorization

$$\begin{array}{ll} {\left\|x-y\right\|}^{2}\geq & \frac{1}{2}{\left\|x_{n}-y_{n}\right\|}^{2}+{\left(x_{n}-y_{n}\right)}^{⊤}\left(x-x_{n}\right)\\ & +\frac{1}{2}{\left\|x_{n}-y_{n}\right\|}^{2}+{\left(y_{n}-x_{n}\right)}^{⊤}\left(y-y_{n}\right)\\ = & {\left\|x_{n}-y_{n}\right\|}^{2}+{\left(x_{n}-y_{n}\right)}^{⊤}\left(x-y-x_{n}+y_{n}\right). \end{array}$$

To improve $\|x-y{\|}^{2}$, we take

xn+1,yn+1∈argmax(x,y)∈S×Sxn-ynyn-xn⊤xy=argmaxx∈Sxn-yn⊤x×argmaxy∈Syn-xn⊤y,

which is a Frank–Wolfe update for the function $f(x,y)=\frac{1}{2}\|x-y{\|}^{2}$ on the Cartesian product S×S.

For the sake of completeness, the Julia functions for our two Frank–Wolfe algorithms follow. The maximum number of iterations, 100, and the convergence tolerances, 10^−8^ and 2 × 10^−8^, can be reset by the user.


function farthest(Supp::Function, x, p)
tol = 1.0e-8
for iter = 1:100
xnew = Supp(x - p)
conv = norm(xnew - x)
x .= xnew
if conv < tol
break
end
end
return (norm(x - p), x)
end
function widest(Supp::Function, x, y)
tol = 2.0e-8
for iter = 1:100
(xnew, ynew) = (Supp(x - y), Supp (y - x))
conv = norm(xnew - x) + norm(ynew - y)
x .= xnew
y .= ynew
if conv < tol
break
end
end
return (norm(x - y), x, y)
end


Projected gradient ascent offers another avenue for solving the farthest and diameter problems. The two algorithms we later derive and apply are

xn+1=PS2xn-pfor;farS(p)xn+1yn+1=PS(32xn-12yn)PS(32yn-12xn)for;diam(S).

As MM algorithms, these two algorithms are guaranteed to increase the objective function. Fortunately, projected gradient ascent does not require the objective function to be convex. Once again the Julia code is straightforward.


function farthest(Proj::Function, x, p)
tol = 1.0e-8
for iter = 1:100
xnew = Proj(2x - p)
conv = norm(xnew - x)
x .= xnew
if conv < tol
break
end
end
return (norm(x - p), x)
end
function widest(Proj::Function, x, y)
tol = 2.0e-8
for iter = 1:100
(xnew, ynew) = (Proj(3x / 2 - y / 2), Proj(3y / 2 - x / 2))
conv = norm(xnew - x) + norm(ynew - y)
x .= xnew
y .= ynew
if conv < tol
break
end
end
return (norm(x - y), x, y)
end


The contributions of this article include (a) deriving and testing these algorithms, (b) investigating simplifications arising from symmetry, (c) describing a homotopy method for avoiding local maxima, (d) finding the support function of the intersection of a convex cone and a ball centered at the origin, and (e) elaborating a known bisection algorithm for calculating the support function of a convex sublevel set. The next section derives the projected gradient ascent algorithm and lays out our contributions to items (b) through (e). Section 3 briefly tackles convergence of the various algorithms. Section 4 describes a few numerical experiments, and Section 5 discusses overall conclusions, limitations, and new directions for research. The author’s earlier article [26] applies similar techniques to the problem of computing the Hausdorff distance between two compact convex sets

Here are the notational conventions used throughout this article. All vectors appear in boldface. All entries of the vector 0 equal 0. The ⊤ superscript indicates a vector transpose. The Euclidean norm of a vector x is denoted by ‖x‖. For a smooth real-valued function f(x), I write its gradient (column vector of partial derivatives) as ∇f(x) and its first differential (row vector of partial derivatives) as $df(x)=\nabla f{\left(x\right)}^{⊤}$. Finally, I denote the directional derivative of f(x) in the direction v by $d_{v}f(x)$. When f(x) is differentiable, $d_{v}f(x)=df(x)v$.

## 2. Derivations

### 2.1. Projected Gradient Ascent and Homotopy

Let us first tackle the closest, farthest, and diameter problems when the projection operator $P_{S}(p)$ is available. These three problems are equivalent to minimizing the functions

$$c(x)=\frac{1}{2}\|x-p{\|}^{2}$$

$$f(x)=-\frac{1}{2}\|x-p{\|}^{2}$$

$$w(x,y)=-\frac{1}{2}\|x-y{\|}^{2}$$

with gradients

∇c(x)=x-p

∇f(x)=-(x-p)

∇w(x,y)=y-xx-y

over the sets S and S×S. One possibility for this task is projected gradient descent.

Because the Lipschitz constants of ∇c(x) and ∇f(x) are both 1, the projected gradient steps for the closest and farthest problems are

$$\begin{array}{ll} x_{n+1} & =P_{S}\left[x_{n}-L^{-1}\nabla c\left(x_{n}\right)\right]\\ & =P_{S}\left(x_{n}-x_{n}+p\right)\\ & =P_{S}(p) \end{array}$$

$$\begin{array}{ll} x_{n+1} & =P_{S}\left[x_{n}-L^{-1}\nabla f\left(x_{n}\right)\right]\\ & =P_{S}\left(x_{n}-p+x_{n}\right)\\ & =P_{S}\left(2x_{n}-p\right). \end{array}$$

As expected, the closest algorithm $x_{n+1}=P_{S}(p)$ reduces to ordinary projection. The Lipschitz constant for ∇w(x,y) is determined by

y-xx-y-v-uu-v=2‖y-x-v+u‖2≤2(‖x-u‖+‖y-v‖)2≤4(‖x-u‖2+‖y-v‖2)=2x-uy-v

as 2. Consequently, the projected gradient step for the diameter problem is

xn+1yn+1=PS×Sxnyn-12∇wxn,yn=PS×Sxnyn-12yn-xnxn-yn=PS(32xn-12yn)PS(32yn-12xn).

This diameter update relies on the facts that minimization of a Euclidean distance is equivalent to minimization of a squared Euclidean distance and that the squared objective splits over the x and y parameters.

The chief problem with all of the proposed algorithms is their propensity to veer toward local maxima. One remedy is good initialization. Given a point p∉S, a general tactic for initialization in the farthest problem is to start with the projection $P_{S}(p)$. This defines the line segment $\left[p,P_{S}(p)\right]$, which can be extended until it hits the boundary of S at a second point $x_{0}$. Replacing $P_{S}(p)$ by $x_{0}$ produces a more distant point of S and a better starting value in the farthest problem. If S is an even set in the sense that S=-S, then as discussed in Section 2.3, the second point reduces to $P_{S}(-p)$.

Beyond good initialization, it is worth considering the expensive alternative of homotopy [27] for the diameter problem. The idea is to gradually deform the unit ball B, where the diameter problem is trivial to solve, into the target set S. Thus, we follow the solution path along the family of sets tS+(1-t)B from t=0 to t=1. For the Frank–Wolfe method, I exploit the fact that the Minkowski convex combination tS+(1-t)B has support map $t\;{supp}_{S}\left(z\right)+\left(1-t\right){supp}_{B}(z)$.

For projected gradient ascent, one can project points onto the Minkowski convex combination tS+(1-t)B by two devices. First, it is well known that $P_{tS}(z)=tP_{S}\left(t^{-1}z\right)$ for any t>0. Second, there is an effective algorithm for projecting onto a Minkowski sum A+B [28]. The idea is to alternate minimization of ‖z-a-b‖ with respect to a∈A and b∈B. The iterative scheme $a_{n+1}=P_{A}\left(z-b_{n}\right)$ and $b_{n+1}=P_{B}\left(z-a_{n+1}\right)$ is guaranteed to converge at a linear rate when either set is strongly convex. In particular, (1-t)B is strongly convex. The homotopy method is motivated by the intuition that the early sets are more rounded and that the objective possesses fewer local maxima. The price for better performance is iterations within iterations and an overall slower algorithm.

The following Julia code implements the homotopy method for the diameter problem under Frank-Wolfe. The map ${supp}_{S}(y)$ is passed to each of the functions. The code for projected gradient ascent is similar.


function MinkowskiSupp(Supp::Function, z, t)
return t × Supp(z)+(1 - t) × (1 / norm(z)) × z
end
function widest_homotopy(Supp::Function, n)
x = randn(n)
x = x /norm(x) # random point on unit sphere
y = −x # point on opposite side of unit sphere
(homotopy_points, tol) = (10, 2.0e-8)
for iter = 0:homotopy_points
t = iter / homotopy_points
for i = 1:100
xnew = MinkowskiSupp(Supp, x - y, t)
ynew = MinkowskiSupp(Supp, y - x, t)
conv = norm(x - xnew) + norm(y - ynew)
x .= xnew
y .= ynew
if conv < tol
break
end
end
end
return (norm(x - y), x, y)
end


### 2.2. Supporting Points and Sublevel Sets

The set of supporting points ${supp}_{S}(v)={argmax}_{x\in S}v^{⊤}x$ determines the support function $\sigma_{S}(v)$. Our numerical tests require knowing ${supp}_{S}(v)$ in some specific examples. For instance, the $\ell_{1}$ unit ball has ${supp}_{S}(v)$ equal to the convex hull of the vertices $\pm e_{i}$ where $\left|v_{i}\right|$ is largest. Here, $e_{i}$ is included when $v_{i}>0$, and $-e_{i}$ is included when $v_{i}<0$. For the unit simplex, ${supp}_{S}(v)$ equals the convex hull of the vertices $e_{i}$ where $v_{i}$ is largest. The rectangle [a,b] is a Cartesian product. Hence, ${supp}_{S}(v)$ is also a Cartesian product. In the one-dimensional case, ${supp}_{S}(v)$ is a when $v_{i}<0,b$ when $v_{i}>0$, and all of [a,b] when $v_{i}=0$. For a Minkowski sum $A+B,\;{supp}_{A+B}(v)={supp}_{A}(v)+{supp}_{B}(v)$. This fact plus the identity ${supp}_{tA}\left(v\right)=t\;{supp}_{A}(v)$ for t≥0 makes it easy to carry out the homotopy method with our Frank-Wolfe algorithms.

If ${supp}_{S}(v)$ is a singleton, then $\sigma_{S}(v)$ is differentiable at v by Danskin’s theorem [7]. Conversely, if $\sigma_{S}(v)$ is differentiable, then ${supp}_{S}(v)$ is a singleton by Corollary 25.3.1 of Rockafellar [29]. Because $\sigma_{S}(v)$ is convex, finite, and locally Lipschits, Rademacher’s theorem [30,31] implies that it is differentiable almost everywhere. Hence, $\sigma_{S}(v)$ is a singleton almost everywhere.

Later, we will need ${supp}_{A}(v)$ for the intersection of the non-negative orthant and the ball $B_{r}$ of radius r around the origin. This is a special case of projection onto the intersection of an arbitrary closed convex cone K and the ball. In this general setting, $w={supp}_{K\cap B_{r}}(v)=\frac{r}{\left\|P_{K}(v)\right\|}P_{K}(v)$ when $P_{K}(v)\neq 0$, and w=0, otherwise. To prove this assertion, it suffices to show that

$$d_{u-w}\sigma_{S}(v)=v^{⊤}(u-w)\leq 0$$

for any point $u\in K\cap B_{r}$. When $P_{K}(v)\neq 0$, the Moreau decomposition and the Cauchy-Schwarz inequality imply that

$$\begin{array}{ll} v^{⊤}(w-u) & ={\left[P_{K}(v)+P_{K^{\circ }}(v)\right]}^{⊤}(w-u)\\ & =\frac{r}{\left\|P_{K}(v)\right\|}{\left\|P_{K}(v)\right\|}^{2}-{\left[P_{K}(v)+P_{K^{\circ }}(v)\right]}^{⊤}u\\ & \geq \frac{r}{\left\|P_{K}(v)\right\|}{\left\|P_{K}(v)\right\|}^{2}-P_{K}{\left(v\right)}^{⊤}u\\ & \geq r\left\|P_{K}(v)\right\|-r\left\|P_{K}(v)\right\|\\ & =0, \end{array}$$

where $K^{\circ }$ is the polar cone of K. Otherwise, $P_{K}(v)=0$, and

$$v^{⊤}(w-u)=-{\left[P_{K}(v)+P_{K^{\circ }}(v)\right]}^{⊤}u=-P_{K^{\circ }}{\left(v\right)}^{⊤}u\geq 0$$

by definition.

The polar cone of the non-negative orthant consists of those vectors v with v≤0. The current intersection support map can also be deduced less directly from Proposition 2.2 of paper [32]. The corresponding problem of projecting onto the intersection of a ball and cone is treated by Lange [7] and Bauschke et al. [33].

As already mentioned, the support function of a sublevel set is generally challenging to compute. As a supplement to the brief discussion in Section 6.4.2 of the survey [12], consider the Lagrangian $-v^{⊤}x+\mu [g(x)-c]$. If g(x) is differentiable, then the Lagrangian satisfies the KKT stationary conditions

0=-v+μ∇g(x)

0=g(x)-c.

Slater’s condition postulates the existence of a point y with g(y)<c. Under this condition, there is μ≥0 satisfying the KKT conditions. The solution is unique and can be found by bisection when v≠0 and g(x) is not just convex but also strongly convex. If h(u) is the inverse function of ∇g(x), then one seeks a root of the equation $\phi (\mu )=g\left[h\left(\mu^{-1}v\right)\right]=c$ by bisection. Recall that the equation ∇g(x)=y is uniquely solvable for all y when g(x) is strongly convex and differentiable. The function ϕ(μ) is strictly decreasing in μ because

$$\begin{array}{ll} \phi^{\prime }(\mu ) & =dg\left[h\left(\mu^{-1}v\right)\right]dh\left(\mu^{-1}v\right)\left(-\mu^{-2}v\right)\\ & =\mu^{-1}v^{⊤}dh\left(\mu^{-1}v\right)\left(-\mu^{-2}v\right)\\ & =-\mu^{-3}v^{⊤}d^{2}g{\left[h\left(\mu^{-1}v\right)\right]}^{-1}v\\ & <0 \end{array}$$

when g(x) is also twice differentiable. Finally, under strong convexity, $\phi^{\prime }(\mu )$ tends to -∞ as μ↓0, so ϕ(μ)→∞ as μ↓0.

As an example, consider the sublevel set {x:g(x)≤c} determined by the elastic net function $g(x)=\|x{\|}_{1}+\frac{\rho }{2}\|x{\|}^{2}$, which is separable but not fully differentiable. There is at least a partial inverse. Because the partial derivative $\frac{\partial }{\partial x_{i}}g(x)=sgn\left(x_{i}\right)+\rho x_{i}$, the i th component of the inverse function h(u) should satisfy

$$u_{i}=\nabla g[h(u){]}_{i}=sgn\left[h(u{)}_{i}\right]+\rho h(u{)}_{i}.$$

Hence, the function h(u) with i th component $h(u{)}_{i}=\frac{sgn\left(u_{i}\right)}{\rho }{\left(\left|u_{i}\right|-1\right)}_{+}$ serves as a partial inverse. For v≠0 and c>0, the function $\phi (\mu )=g\left[h\left(\mu^{-1}v\right)\right]$ is continuous, tends to 0 as μ→∞ and to ∞ as μ→0. Hence, the equation ϕ(μ)=c is solvable by the intermediate value theorem.

### 2.3. Symmetry

Let us first consider a permutationally invariant set S. In S, we can swap coordinates and remain within S. For a support function $x={supp}_{S}(y)$, the swap criterion dictates that

$$\begin{array}{rr} y_{i}x_{j}+y_{j}x_{i} & \leq y_{i}x_{i}+y_{j}x_{j}⟺\\ 0 & \leq \left(y_{i}-y_{j}\right)\left(x_{i}-x_{j}\right). \end{array}$$

Thus, the components of the map $x={supp}_{S}(y)$ should satisfy the inequality

$$\left(y_{i}-y_{j}\right)\left(x_{i}-x_{j}\right)\geq 0$$

for all i and j. In other words, the components of the output $x={supp}_{S}(y)$ should be consistently ordered with the components of the input y of ${supp}_{S}(y)$.

For the distance function, the swap criterion is

$$\begin{array}{rr} {\left(y_{i}-x_{j}\right)}^{2}+{\left(y_{j}-x_{i}\right)}^{2} & \geq {\left(y_{i}-x_{i}\right)}^{2}+{\left(y_{j}-x_{j}\right)}^{2}⟺\\ 0 & \leq \left(y_{i}-y_{j}\right)\left(x_{i}-x_{j}\right). \end{array}$$

Hence, the components of the output $x=P_{S}(y)$ should also be consistently ordered with the components of the input y of $P_{S}(y)$.

For the farthest function, the swap criterion is

$$\begin{array}{rr} {\left(y_{i}-x_{j}\right)}^{2}+{\left(y_{j}-x_{i}\right)}^{2} & \leq {\left(y_{i}-x_{i}\right)}^{2}+{\left(y_{j}-x_{j}\right)}^{2}⟺\\ 0 & \geq \left(y_{i}-y_{j}\right)\left(x_{i}-x_{j}\right). \end{array}$$

Hence, the components of the map $x={far}_{S}(y)$ should satisfy the inequality

$$\left(y_{i}-y_{j}\right)\left(x_{i}-x_{j}\right)\leq 0$$

for all i and j. In other words, the components should be reverse consistently ordered.

With each of these three functions, permutational invariance should inform our choice of algorithm initial points. Alternatively, if one opts to test the extreme points of a set S to find ${far}_{S}(y)$, then most of these can be eliminated from contention by incompatibility with permutational invariance. A sublevel set {x:g(x)≤c} is permutationally invariant when g(x) is permutationally invariant.

Finally, let us turn to the diameter problem for even sets S=-S. Choose x∈S farthest from the origin. The Cauchy-Schwarz inequality $-y^{⊤}x\leq \|x{\|}^{2}$ for y∈S implies that

$$\begin{array}{rr} \|y{\|}^{2}-2y^{⊤}x & \leq 3{\left\|x\right\|}^{2}⟺\\ \|y-x{\|}^{2} & \leq 4{\left\|x\right\|}^{2}=\|(-x)-x{\|}^{2}. \end{array}$$

Thus, the diameter 2‖x‖ is achieved when ‖x‖ is maximal. For instance, the diameter of the elastic net sublevel set $\left\{x\;:\|x{\|}_{1}+\frac{1}{2}\|x{\|}^{2}\leq 1\right\}$ is $2(\sqrt{3}-1)$, regardless of the dimension. The maximum value $\sqrt{3}-1=0.73205$ of ‖x‖ is achieved when all but one component of x is 0. Sometimes a translate S+b of S is even when S itself is not even. If this is the case, the diameter can be found by maximizing ‖x+b‖ over S. A sublevel set {x:g(x≤c} is even when g(x) is an even function.

## 3. Convergence

Zangwill’s [34] theorem offers the quickest route to proving convergence of both projected gradient ascent and our simplified Frank-Wolfe algorithm. Unfortunately, Zangwill’s theorem says nothing about the rate of convergence. The theorem involves a solution set Γ⊂S, a descent function f(x), and an algorithm map M(x). In our case, Γ consists of the stationary points x∈S where $d_{v}f(x)\geq 0$ for all tangent vectors v. Note that this condition is necessary but not sufficient for x to be a local minimum of f(x). The algorithm map M(x) is said to be closed at x if whenever $x_{n}\in S$ converges to x and $y_{n}\in M\left(x_{n}\right)$ converges to y, then y∈M(x). If M(z) is single-valued and continuous at x, then M(z) is certainly closed at x. Here is Zangwill’s theorem.

### Proposition 1.

*Suppose that*

1. *All iterates* $x_{n+1}\in M\left(x_{n}\right)$ *fall in the compact set* S.
2. *The map* M *is closed at* x *when* x∉Γ.
3. *The function* f(x) *is continuous on* S *and satisfies* f[M(x)]≤f(x), *with strict inequality for* x∉Γ.

*Then, the limit of any convergent sub-sequence* $x_{n_{m}}$ *of* $x_{n}$ *belongs to* Γ.

Zangwill’s theorem also applies to maximization provided that the progress condition f[M(x)]≤f(x) is replaced by f[M(x)]≥f(x), and the stationary condition $d_{v}f(x)\geq 0$ is replaced by $d_{v}f(x)\leq 0$.

### 3.1. Convergence of Frank–Wolfe

To prove that the algorithm map is closed for our version of Frank–Wolfe, suppose that $df\left(x_{n}\right)y_{n}\geq df\left(x_{n}\right)y$ for every y∈S and that $x_{n}$ and $y_{n}$ converge to $x_{\infty }$ and $y_{\infty }$. Then assuming df(x) is continuous, $df\left(x_{\infty }\right)y_{\infty }\geq df\left(x_{\infty }\right)y$ for every y∈S. The ascent property is baked into Frank-Wolfe because it is a minorization-maximization algorithm. Finally, a stationary point x satisfies df(x)v≤0 for all tangent vectors v. The set of tangent vectors v is the closure of the set of points c(y-x) with y∈S and c>0. This is where the convexity of S comes into play. Hence, x is a stationary point if and only if df(x)x≥df(x)y for all y∈S, which is equivalent to x∈M(x). If x∉M(x), then the objective strictly increases. Thus, Zangwill’s theorem applies to Frank-Wolfe.

These arguments shed light on the rate of convergence as measured by closeness to stationarity [24,35]. Indeed, adding the inequality

$$f\left(x_{n+1}\right)-f\left(x_{n}\right)\geq df\left(x_{n}\right)\left(x_{n+1}-x_{n}\right)\geq df\left(x_{n}\right)\left(y-x_{n}\right)$$

for arbitrary y∈S leads by telescoping to

$$(n+1)\underset{0\leq k\leq n}{min}\underset{y\in S}{max}\;df\left(x_{k}\right)\left(y-x_{k}\right)\leq f\left(x_{n+1}\right)-f\left(x_{0}\right).$$

This in turn implies

$$\underset{0\leq k\leq n}{min}\underset{y\in S}{max}\;df\left(x_{k}\right)\left(y-x_{k}\right)\leq \frac{1}{n\;+\;1}\left[\underset{x\in S}{max}\;f(x)-f\left(x_{0}\right)\right].$$

Thus, the stationary condition ${max}_{y\in S}\;df(x)(y-x)\leq 0$ is reasonable to expect at a limit point x of Frank–Wolfe.

Mangasarian [36] demonstrates that Frank–Wolfe converges in a finite number of iterations when the objective f(x) is differentiable, convex, and bounded from above on a polyhedral set S. This result is more or less obvious given that the number of extreme points of S is finite. Yurtsever and Sra [37] show that the well-known convex–concave procedure (СССР) [23] and its generalization are special cases of the Frank–Wolfe method with step-size selection. This insight quantifies the convergence rate of the CCCP.

### 3.2. Convergence of Projected Gradient Descent

In this scenario, let f(x) be the function to be minimized. Furthermore, let L be the Lipschitz constant of ∇f(x) on S. Projected gradient descent minimizes the surrogate function $g\left(x|x_{n}\right)=f\left(x_{n}\right)+df\left(x_{n}\right)\left(x-x_{n}\right)+\frac{L}{2}{\left\|x-x_{n}\right\|}^{2}$ over S. The algorithm map

$$x_{n+1}=M\left(x_{n}\right)=P_{S}\left[x_{n}-L^{-1}\nabla f\left(x_{n}\right)\right]$$

is single-valued and continuous. The descent condition is again automatic. Proposition 7.3.2 of [7] now shows that Zangwill’s theorem applies provided that we identify stationary points as satisfying either of the equivalent conditions M(x)=x or f[M(x)]=f(x). These conditions are also equivalent to our postulated stationary condition because

$$\nabla g\left(x_{n}|x_{n}\right)=\nabla f\left(x_{n}\right).$$

In this regard, observe that $g\left(x|x_{n}\right)$ is strongly convex, so the requirement $dg\left(x_{n}|x_{n}\right)v\geq 0$ for all tangent vectors v is both necessary and sufficient for $x_{n}$ to be a global minimum of $g\left(x|x_{n}\right)$. Proposition 7.3.4 of my book [7] additionally proves that the collection of limit points of the MM sequence $x_{n+1}=M\left(x_{n}\right)$ is compact and connected. Thus, when the extreme points of S are isolated, the MM sequence actually converges to one of them. Alternatively, in the setting of semi-algebraic sets and functions, Attouch et al. [38] prove full convergence using the tools of algebraic geometry.

To attack the rate of convergence of non-convex projected gradient descent, I present a simplified version of an argument featured by Beck [5]. The references [6,35,39–41] provide further background. Our point of departure is the observation that the stationary condition df(x)(y-x)≥0 for all y∈S is equivalent to satisfaction of the equation $x=P_{S}[x-s\nabla f(x)]$ for any s>0. The obtuse angle criterion

$${\left[x-s\nabla f(x)-x\right]}^{⊤}(y-x)\leq 0$$

for all y∈S is both necessary and sufficient for $x=P_{S}[x-s\nabla f(x)]$. However, the obtuse angle criterion is just a disguised version of df(x)(y-x)≥0 for all y∈S.

The quantity $\left\|x_{n}-P_{S}\left[x_{n}-L^{-1}\nabla f\left(x_{n}\right)\right]\|=\|x_{n}-x_{n+1}\right\|$ serves as a measure of how far $x_{n}$ is from stationarity. The obtuse angle condition implies that

$${\left[x_{n}-L^{-1}\nabla f\left(x_{n}\right)-x_{n+1}\right]}^{⊤}\left(x_{n}-x_{n+1}\right)\leq 0,$$

which is equivalent to

$$df\left(x_{n}\right)\left(x_{n+1}-x_{n}\right)\leq -L{\left\|x_{n}-x_{n+1}\right\|}^{2}.$$

It follows that

$$\begin{array}{ll} f\left(x_{n+1}\right)-f\left(x_{n}\right) & \leq g\left(x_{n+1}|x_{n}\right)-g\left(x_{n}|x_{n}\right)\\ & \leq dg\left(x_{n}|x_{n}\right)\left(x_{n+1}-x_{n}\right)+\frac{L}{2}{\left\|x_{n+1}-x_{n}\right\|}^{2}\\ & =df\left(x_{n}\right)\left(x_{n+1}-x_{n}\right)+\frac{L}{2}{\left\|x_{n}-x_{n+1}\right\|}^{2}\\ & \leq -L{\left\|x_{n+1}-x_{n}\right\|}^{2}+\frac{L}{2}{\left\|x_{n}-x_{n+1}\right\|}^{2}. \end{array}$$

Rearrangement of this inequality gives

$${\left\|x_{n+1}-x_{n}\right\|}^{2}\leq \frac{2}{L}\left[f\left(x_{n}\right)-f\left(x_{n+1}\right)\right].$$

Adding these inequalities and telescoping yield

$$\begin{array}{ll} \underset{0\leq k\leq n}{min}\left\|x_{n+1}-x_{n}\right\| & \leq \sqrt{\frac{1}{n\;+\;1}\sum_{0\leq k\leq n}{\left\|x_{n+1}-x_{n}\right\|}^{2}}\\ & \leq \sqrt{\frac{2}{L(n\;+\;1)}\left[f\left(x_{0}\right)-\underset{x\in S}{min}f(x)\right]}. \end{array}$$

In other words, the convergence rate is $O(\frac{1}{\sqrt{n}})$ for the distance from stationarity.

## 4. Numerical Experiments

We tested the Frank–Wolfe and projected gradient ascent algorithms on five compact convex sets: (a) the box $[-1,1]=\left\{x\;:\|x{\|}_{\infty }\leq 1\right\}$, (b) the intersection of the unit ball and the non-negative orthant, (c) the probability simplex, (d) the $\ell_{1}$ unit ball, and (e) the sublevel set $\left\{x\;:{\left\|x\right\|}_{1}+\frac{1}{2}{\left\|x\right\|}^{2}\leq 1\right\}$ determined by the elastic net penalty. All five sets are permutationally invariant. Sets (a), (d), and (e) are even. These examples are representative, and all five exact diameters are available for comparison. Tables 1 and 2 present our findings. The column headed “Fraction” records the fraction of the 100 trials that achieve the maximum objective. The column headed “Seconds” records cumulative execution times over 100 trials.

Homotopy offers no advantage in the farthest problem and is limited to the diameter problem. The farthest problem is initialized by -p under Frank-Wolfe and by $P_{C}(-p)$ under projected gradient ascent. Thus, all 100 trials are identical in each scenario. The diameter problem is initialized by two random opposing points on the unit sphere. Given the nature of the support and projection maps, all subsequent iterations fall within the set C. Both the Frank-Wolfe algorithms and the projected gradient descent algorithms perform well. They attain the same solutions, which in the diameter problems are identical to the known solution. Contrary to our expectation, projected gradient ascent is not noticeably slower than Frank–Wolfe. Homotopy over 11 intervening points rescues projected gradient descent on the diameter problem for sets (b) and (c). Homotopy is noticeably slower than the unadorned algorithms. The elastic net problem tends to take the most time owing to the inefficiency of bisection. Computation times scale well when the dimension d of the ambient space reaches 1000. All computations were carried out on a MacBook Pro with a 2.3 GHz 8 -core i9 chip and 16 GB of memory. Although the algorithms are embarrassingly parallel across trials, our Julia code is completely serial.

On the basis of reliability, these problems favor Frank–Wolfe. Although Frank–Wolfe algorithms possess a theoretically faster rate of convergence than projected gradient ascent, in the empirical trials, the two methods are quite comparable in speed. The “Seconds” columns of Tables 1 and 2 have respective means of 0.260 and 0.240 and respective medians of 0.065 and 0.038. The corresponding standard deviations 1.192 and 0.697 are relatively high, so for this and other obvious reasons, one should probably not read too much into these crude comparisons.

## 5. Discussion

The farthest and diameter problems are natural problems of intrinsic interest. Given their non-convexity, they have not received nearly the attention in the mathematical literature as the closest problem. Exact mathematical solutions are available in some special cases. Research on fast algorithms appears to be limited to random point clouds. Infinite sets defined by mathematical formulas have been largely ignored. The current paper partially rectifies this omission and demonstrates the value of continuous optimization techniques. The Frank–Wolfe and projected gradient ascent algorithms are relatively easy to code and extremely fast in high dimensions. Our preliminary experiments tilt toward the Frank–Wolfe algorithms as the more reliable of the two options. The full Julia code for our numerical examples appears at https://github.com/KennethLange/ClosestFarthestWidest (accessed on 19 January 2024).

Standard convergence arguments covered here guarantee that all limit points of the two algorithm classes are stationary points. I suspect, but have not proven, that full convergence to a stationary point always occurs for the Frank–Wolfe algorithms. This exercise would require a foray into the difficult terrain of real algebraic geometry [42]. In any event, convergence to a global maximum is not guaranteed. Fortunately, safeguards can be put in place to improve the chances of successful convergence. I have suggested symmetry tactics for choosing good starting points. The homotopy method capitalizes on exact solutions for the unit ball. Minkowski set rounding smooths the boundary of the target set and steers iterates in a productive direction. For semi-algebraic sets and functions, Attouch et al. [38] prove that the projected gradient algorithms always converge for objectives like ours with Lipschitz gradients. Again, convergence to a global maximum is not guaranteed.

I hope that this paper will provoke greater focus on the farthest and diameter problems. As prototype non-convex problems, they are worthy of further attention. In view of the connections to other Frank–Wolfe algorithms, I also encourage more community effort in finding and cataloging support functions $\sigma_{S}(y)$ and their corresponding maps ${supp}_{S}(v)$. The effort put into this task so far is weaker than the effort put into devising projection maps. Once in possession of such maps, construction of fast algorithms is immensely easier. Finally, I would like to highlight the illumination that the MM principle brings to the construction of new high-dimensional optimization algorithms, including the ones considered here.

## Figures and Tables

**Table 1.** Evaluation of the Frank–Wolfe algorithms.

Set	Dimension	Type	Homotopy	Fraction	Maximum	Seconds

box	2	farthest	no		2.9624	0.13
box	2	widest	no	1.0	2.8284	0.0708
box	2	widest	yes	1.0	2.8284	0.0709
ball ∩ orthant	2	farthest	no		2.5994	0.0817
ball ∩ orthant	2	widest	no	0.52	1.4142	0.0756
ball ∩ orthant	2	widest	yes	0.52	1.4142	0.0612
simplex	2	farthest	no		2.5465	0.0734
simplex	2	widest	no	1.0	1.4142	0.0697
simplex	2	widest	yes	1.0	1.4142	0.0707
L1 ball	2	farthest	no		2.5465	0.0754
L1 ball	2	widest	no	1.0	2.0	0.0764
L1 ball	2	widest	yes	1.0	2.0	0.0761
elastic net	2	farthest	no		2.2883	0.113
elastic net	2	widest	no	1.0	1.4641	0.0818
elastic net	2	widest	yes	1.0	1.4641	0.277
box	3	farthest	no		3.9572	0.0472
box	3	widest	no	1.0	3.4641	0.0458
box	3	widest	yes	1.0	3.4641	0.0651
ball ∩ orthant	3	farthest	no		3.2791	0.0443
ball ∩ orthant	3	widest	no	0.78	1.4142	0.0436
ball ∩ orthant	3	widest	yes	0.78	1.4142	0.0694
simplex	3	farthest	no		3.0727	0.045
simplex	3	widest	no	1.0	1.4142	0.0461
simplex	3	widest	yes	1.0	1.4142	0.0763
L1 ball	3	farthest	no		3.0727	0.0439
L1 ball	3	widest	no	1.0	2.0	0.0465
L1 ball	3	widest	yes	1.0	2.0	0.0671
elastic net	3	farthest	no		2.8509	0.0705
elastic net	3	widest	no	1.0	1.4641	0.0489
elastic net	3	widest	yes	1.0	1.4641	0.249
box	10	farthest	no		5.6758	0.0475
box	10	widest	no	1.0	6.3246	0.0454
box	10	widest	yes	1.0	6.3246	0.0612
ball ∩ orthant	10	farthest	no		3.6022	0.043
ball ∩ orthant	10	widest	no	1.0	1.4142	0.0449
ball ∩ orthant	10	widest	yes	1.0	1.4142	0.0848
simplex	10	farthest	no		3.2937	0.0449
simplex	10	widest	no	1.0	1.4142	0.0442
simplex	10	widest	yes	1.0	1.4142	0.0797
L1 ball	10	farthest	no		3.2937	0.0434
L1 ball	10	widest	no	1.0	2.0	0.0427
L1 ball	10	widest	yes	1.0	2.0	0.0696
elastic net	10	farthest	no		3.1077	0.0496
elastic net	10	widest	no	1.0	1.4641	0.0537
elastic net	10	widest	yes	1.0	1.4641	0.328
box	1000	farthest	no		59.61	0.0444
box	1000	widest	no	1.0	63.246	0.0505
box	1000	widest	yes	1.0	63.246	0.175
ball ∩ orthant	1000	farthest	no		31.962	0.0425
ball ∩ orthant	1000	widest	no	1.0	1.4142	0.0447
ball ∩ orthant	1000	widest	yes	1.0	1.4142	0.469
simplex	1000	farthest	no		31.325	0.0456
simplex	1000	widest	no	1.0	1.4142	0.046
simplex	1000	widest	yes	1.0	1.4142	0.548
L1 ball	1000	farthest	no		31.325	0.0427
L1 ball	1000	widest	no	1.0	2.0	0.0445
L1 ball	1000	widest	yes	1.0	2.0	0.436
elastic net	1000	farthest	no		31.3	0.523
elastic net	1000	widest	no	1.0	1.4641	0.277
elastic net	1000	widest	yes	1.0	1.4641	9.21

**Table 2.** Evaluation of the projected gradient ascent algorithms.

Set	Dimension	Type	Homotopy	Fraction	Maximum	Seconds

box	2	farthest	no		2.9624	0.802
box	2	widest	no	1.0	2.8284	0.0291
box	2	widest	yes	0.99	2.8284	0.129
ball ∩ orthant	2	farthest	no		2.5994	0.128
ball ∩ orthant	2	widest	no	0.78	1.0	0.0278
ball ∩ orthant	2	widest	yes	0.51	1.4142	0.09
simplex	2	farthest	no		2.5465	0.761
simplex	2	widest	no	1.0	1.4142	0.0259
simplex	2	widest	yes	0.7	1.4142	0.0906
L1 ball	2	farthest	no		2.5465	0.154
L1 ball	2	widest	no	1.0	2.0	0.0273
L1 ball	2	widest	yes	0.99	2.0	0.0912
elastic net	2	farthest	no		2.2883	0.159
elastic net	2	widest	no	1.0	1.4641	0.0279
elastic net	2	widest	yes	0.82	1.4641	0.112
box	3	farthest	no		3.9572	0.0269
box	3	widest	no	1.0	3.4641	0.0246
box	3	widest	yes	0.99	3.4641	0.0357
ball ∩ orthant	3	farthest	no		3.2791	0.0257
ball ∩ orthant	3	widest	no	0.9	1.0	0.0259
ball ∩ orthant	3	widest	yes	0.77	1.4142	0.0286
simplex	3	farthest	no		3.0727	0.0248
simplex	3	widest	no	0.64	1.4142	0.0258
simplex	3	widest	yes	0.93	1.4142	0.0291
L1 ball	3	farthest	no		3.0727	0.0255
L1 ball	3	widest	no	1.0	2.0	0.025
L1 ball	3	widest	yes	1.0	2.0	0.0296
elastic net	3	farthest	no		2.8509	0.0574
elastic net	3	widest	no	1.0	1.4641	0.0303
elastic net	3	widest	yes	0.74	1.4641	0.0616
box	10	farthest	no		5.6758	0.0283
box	10	widest	no	1.0	6.3246	0.0314
box	10	widest	yes	0.95	6.3246	0.0401
ball ∩ orthant	10	farthest	no		3.6022	0.0268
ball ∩ orthant	10	widest	no	1.0	1.0	0.0279
ball ∩ orthant	10	widest	yes	1.0	1.4142	0.0396
simplex	10	farthest	no		3.2937	0.0272
simplex	10	widest	no	0.08	1.0801	0.0271
simplex	10	widest	yes	0.88	1.4142	0.0327
L1 ball	10	farthest	no		3.2937	0.0271
L1 ball	10	widest	no	1.0	2.0	0.0272
L1 ball	10	widest	yes	0.97	2.0	0.0363
elastic net	10	farthest	no		3.1077	0.0415
elastic net	10	widest	no	1.0	1.4641	0.0289
elastic net	10	widest	yes	0.44	1.4641	0.0962
box	1000	farthest	no		59.61	0.0515
box	1000	widest	no	1.0	63.246	0.0914
box	1000	widest	yes	0.01	63.019	1.11
ball ∩ orthant	1000	farthest	no		31.962	0.0284
ball ∩ orthant	1000	widest	no	1.0	1.0	0.0377
ball ∩ orthant	1000	widest	yes	1.0	1.4142	0.209
simplex	1000	farthest	no		31.325	0.0466
simplex	1000	widest	no	0.02	1.0005	0.0588
simplex	1000	widest	yes	0.02	1.4142	0.632
L1 ball	1000	farthest	no		31.325	0.0435
L1 ball	1000	widest	no	1.0	2.0	0.0722
L1 ball	1000	widest	yes	0.52	2.0	0.566
elastic net	1000	farthest	no		31.3	0.609
elastic net	1000	widest	no	1.0	1.4641	0.29
elastic net	1000	widest	yes	0.03	1.4641	4.2
